# Health Promotion and Identity Construction in Norwegian Kindergartens – A Qualitative Study on Children with and without Disabilities

**DOI:** 10.1007/s10643-022-01382-7

**Published:** 2022-10-10

**Authors:** Ingvild Åmot, Borgunn Ytterhus

**Affiliations:** 1 Queen Maud University College, Trondheim, Norway; 2 Norwegian University of Science & Technology, Department of Public Health and Nursing, Trondheim, Norway

**Keywords:** kindergarten, public health, physical activities, self-construction, well-being

## Abstract

This article focuses on how children, independent of abilities, create healthy identities and spaces in kindergarten, and is based on a qualitative CGT-study carried out in Norwegian Kindergartens. Data sources include Life-form interviews with 24 children, with and without disabilities. Children placed health in the context of their daily-life experiences. They underlined the importance of aspiring to social well-being and creating healthy spaces through internally driven physical exertion and child-controlled activities. Children described how they relate to the staff’s restrictions and legal requirements as “the staff” versus “we, the children”. They advocate for the staff’s engagement in play and activities.

**Supplementary Information:**

The online version contains supplementary material available at 10.1007/s10643-022-01382-7.

## Introduction

By taking a closer look into the everyday life of children in kindergarten, this article illustrates how public-health policy manifests itself in the way children present their everyday experiences. The aim is to add insight into how children create healthy identities and spaces in kindergarten, regardless of their abilities.

Health and wellbeing for all is one of the United Nations Sustainable Develop Goals (SDG). The level of primary health care has been emphasized strongly since the Alma Ata Declaration on Public Health (World Health Organization, 1978). It claims that health is a “human right and that the attainment of the highest possible level of health is a most important world-wide social goal whose realization requires the action of many other social and economic sectors in addition to the health sector”. This includes the education sector where kindergartens constitute the first step in the lifelong education sector learning path. The overriding goal for kindergartens is to educate children through knowledge acquisition and socialization, which is important input for their identity construction (Ministry of Education, 2005). According to the Norwegian Kindergarten Act, Sect. 2, kindergartens in Norway must have a health-promoting and preventive function.

In Norway, public health work is defined in the Act relating to Public Health, Sect. 3 (Ministry of Health and Care, 2012) as:

“…society’s efforts to influence factors that directly or indirectly promote the health and well-being of the population; prevent mental and somatic illnesses, disorders or injuries; or that protect against health threats; as well as efforts seeking a more equal distribution of factors that directly or indirectly affect health”.

The Act highlights and emphasizes that the health of all citizens is a political issue and the responsibility of the public authorities (Ministry of Health & Care, 2012; Mæland 2016). Public health includes both health promotion and health prevention, and consequently operates within two different scientific traditions: the societal and the medical. On the one hand, health promotion refers to an understanding of health as a state of wellbeing and identity-strengthening processes, and on the other hand, health means the opposite of illness/injuries/suffering. This article reports on a qualitative study on health promotion from a social-science point of departure on the micro-level, based on what children themselves tell and do in the social spaces on their identity-construction path.

### Underpinning Theoretical Perspectives

We find theoretical support in the sociologist Aron Antonovsky’s (1987) studies of the origin of health (salutogenesis). He describes health as a dynamic state of being and developing through the construction of a Sense of Coherence (SOC). SOC tells us the degree to which an individual is able to deal with their life and find it comprehensible, manageable, and meaningful, and where meaning-making is highly important. Health promotion is defined by the World Health Organization (WHO, 1998) as “…the process of enabling people to increase control over, and to improve their health.” Moving towards a SOC and meaning-making is an important aspect of health promotion and will be addressed in this article.

Public health work takes place at the political, structural, and social-space levels. This article focuses on the social-space level. When it comes to health prevention, Mæland (2020a, p. 24) describes “social space” as the environment we live in and interactions we experience that influence our robustness and resilience and our risk of illness or death. In this study we have slightly refined Mæland’s definition by claiming that *place* refers to the physical environment whereas *space* refers to the social practices, processes, and interactions taking place in these environments (Ytterhus & Åmot, 2021).

The point of departure of the study positions children as competent and vulnerable actors, which creates social space within the places available for interaction and identity construction. Healthy identity construction is closely related to a high level of SOC, but children’s meaning-making may be present in their social action without necessarily being part of a conscious process or verbal articulation. It may be spontaneous and not necessarily predictable, which gives associations to the social psychologist George Herbert Mead’s (1934) theory of identity construction through the Self. He separates the Self in the *I* and the *Me*. The *I* is the spontaneous acting part of ourselves. The *I* could surprise ourselves by our actions. The *I* is the individual’s action towards the social situation within her/his conduct. The *Me* is “the organized set of attitudes of others which one himself assumes” (Mead, 1934, p. 175); these attitudes emerge in social situations. They are relational and represent the conventional, the habitual, and the basis for what the child reacts to (Mead, 1998, pp. 33–34). The *Me* is the way action arises as an experience, including the normative attitudes of the others and the context. The *I* and the *Me*, important ingredients in the children’s meaning-making processes, may differ in strength depending on the child’s maturity and developmental status.

## Current State of Research on Health Promotion and Prevention in Kindergartens

Epidemiology is the doctrine on the spread of diseases. The assumption in social epidemiology is that the spread of diseases in a society reveals how social privileges and disadvantages are distributed in the population (Mæland 2000a: 24). Consequently, social relations and interaction influence our risk of becoming ill and dying (Mæland, 2020b). Strong and preferred social networks promote health, while unwanted, weak, or a lack of social networks make us less healthy. Some research even contends that the risk-effect of a weak social network on illness and death is stronger than the risk from having a low physical activity level (Holt-Lundstad et al., 2010) ; Mæland 2020b). Holt-Lundstad and colleagues. 2010) found that social relations have a strong impact on health and illness and called for health workers and educators to raise this as an important issue in their health promotion work. According to health definitions, young children (4–5 years of age) perceive health as a multidimensional construct, largely related to being involved, in other words, being able to perform preferred activities and participate in a supportive everyday context (Almqvist, Hellnäs et al., 2006; Stirrup, 2018). They are able to distinguish between several aspects of health from an experienced-based perspective related to everyday experiences, knowing that not having the possibility to perform activities and participate together with others undermines health. Three dimensions serve as main organizers of children’s conceptions of health (Normandeau et al., 1998): being functional; adherence to a good lifestyle, such as a healthy diet and doing physical activities; and a general sense of wellbeing and good relationships with others. Children seemed to place health in the context of their daily-life experiences. Health promotion has an interdependence between individuals and subsystems (Green et al., 1996) that has to be contextualized. Physical activity, social interactions, play, the physical environment, and the beliefs and behavior of the staff affect children’s wellbeing and levels of physical activity and, thereby, affect children’s health situations. Research on health promotion in kindergartens includes studies on such subjects as obesity (Klein et al., 2015; Kobel et al., 2017; Lloyd et al., 2017), nutrition (Frerichs et al., 2016), and physical activity (Sando, 2019a, 2019b; Sando & Mehus, 2019; Wagner et al., 2005). Physical activity of at least moderate to vigorous intensity seems to be consistently favorably associated with multiple health indicators for children between three and four years of age in kindergartens (Carson et al., 2017). The physical environment, available materials, common activities, and the opportunity to influence their everyday lives are also important factors for children’s wellbeing in kindergartens (Sandseter & Seland, 2016). Children express security when they are allowed to influence their situations. Relations, both with other children and with the staff are important for children (Sandseter & Seland, 2017). Caregivers’ interactions with children, their stability and sensitivity have been found to be important aspects for children’s wellbeing (Sando & Mehus, 2019). The staff’s role within physical outdoor settings is significant. The beliefs and behaviors of the staff seem to be the strongest non-architectural, invisible barriers to active physical play (Cammisa et al., 2011). Even though there is some documentation of young children’s understanding of health, we still lack knowledge on health and healthy living in kindergartens where the huge diversity of children’s perspectives across abilities is part of the knowledge production, as will be reported in the following. The article will address the following research question:

How do children with and without disabilities create healthy identity and spaces in kindergarten?

We will start with a description of the kindergarten context in Norway, followed by the methodological section. Later in the text we will present findings from our data material and then discuss them in light of the underpinning theoretical perspectives.

## Background: The Norwegian Context

Since 2006 all children in Norway, from the age of one year, have a legal right to attend kindergarten (The Kindergarten Act, 2005 – last revised 2017, Sect. 12 a). At the same time, the public authorities transferred responsibility for kindergartens from the Ministry of Children and Family Affairs to the Ministry of Education, which led to a shift in focus from care to education. Since then, there has been a large increase in newly established kindergartens, but less attention has been paid to the quality of the educational programs and what kind of childhood is formed. In 2020, a total of 92.8% of all children aged one to five were attending kindergarten (Statistics Norway, 2020). This does not only represent an increase in the number of children attending, but also an increase in diversity and the variety of abilities within the groups of children. In 2017, the public authorities introduced a new Kindergarten Framework Plan (KFP) to secure acceptable education for all children (Norwegian Directorate of Education and Training, 2017). One of the six values in the plan is life skills and health. The health subject has been operationalized to: “Body, movement, food and health”, which are concrete areas of focus. The staff’s responsibility to be familiar with and practice the national guidelines for health promotion and prevention is highlighted: “The kindergarten must promote bodily enjoyment, enjoyment of food and food culture, mental and social wellbeing and physical and mental health” (Norwegian Directorate for Education and Training, 2017, p 49). This reflects a clear health-promotion profile, which represents the public health pillar that is relevant for our study.

## Method

This article reports from a qualitative cross-sectional multi-method study design that was used in Norwegian Kindergartens before the COVID-19 pandemic and therefore had no restrictions on social distance or interactions. The researchers (authors) are both senior researchers and hold respectively a professorate in special education and in health science.

### Design, Sample and Recruitment

The study is based on a strategic sample of 24 children (four to five years of age, 11 girls and 13 boys) from six kindergartens in rural and urban areas in Norway. The kindergartens differ in size and organizational structure: four medium-sized kindergartens (46–79 children), one small (< 45 children), and one large (> 80 children) kindergarten. The children in the study were recruited stepwise: First, we recruited children diagnosed with disabilities that potentially can lead to social difficulties/children with no diagnosis but with the basic understanding of parents and staff that they have special-education needs. As soon as we had consent from parents’ of those children, we recruited children without disability/special-education needs in the same kindergartens. In both groups, we chose children with enough verbal language to enter into a group dialogue, and when possible, we matched the children with and without disabilities according to age and gender. The head of the kindergartens recruited children with disabilities in line with our recruitment criteria. The kindergartens’ staff recommended and recruited the composition of the groups of children without disabilities. All the involved parents received written information from us, and signed a consent form. We have no information about families who refused to participate. Eight of the total of 24 children included have either cognitive, sensory, linguistic, emotional or behavioral difficulties (two girls and six boys). There were 118 full-time equivalents in these departments and in total 405 children in these kindergartens.

### Organization in Research Groups

We told the children about our five planned weekly meetings with each group, consisting of the two authors and on average four children with and without disabilities: in total six groups – one at each kindergarten. Only in one of the groups did one child have a special teacher present in the group meetings. The collaboration with the children in the groups followed recommendations from the Norwegian Children’s Ombud, and the research methods were chosen by the researchers based on their earlier experiences of conducting research with children (Barneombudet, 2013). We started and ended all group meetings with play-based procedures and information about who we were, what our intention with the study was (to find out more about how children experience their life in kindergarten), and the plan for the specific day. Together with the children we conducted:

- Individual drawings of activities and play materials in kindergarten.
- Group drawings of their kindergarten.
- Individual guided outdoor and indoor tours, including digital photos of the best, the most boring, and the scariest places. During these tours our role as researchers was to act as participant observers who did not initiate any activities, only responding to the children’s initiatives, questions, and comments in a field-work manner.
- Group dialogues based on the children’s own questions using a Duplo® tableau combined with Individual Play-Based (IPBI) (Åmot, 2014) and Life-form Interviews (LFI) (Andenæs, 1991).

We also had short, structured dialogues with one of the educators at each department to obtain some background information on pedagogical and organizational aspects. This article reports from 24 individual Life-form Interviews (LFI) combined with an Individual Play-Based interview using a Duplo®-block tableau (IPBI). In LFI, focus is on a detailed description of the day in as concrete and detailed a way as possible (Andenæs, 1991). *Time* is essential as an organizational principle, where the intention was to obtain a chronological picture of children’s experiences in the kindergarten environment. The interviews varied in length, most were between 30 and 40 min long.

Based on the principles for LFI, each child was offered the possibility to express knowledge about everyday actions by building the kindergarten and playing out significant situations from everyday life using and manipulating Duplo®-blocks and figures. Using figures like these can encourage children’s participation in communication (Ahlcrona, 2012). By focusing on concrete situations in this way, children can activate complex and abstract themes, and it is easier to communicate with them on relations, events and the structure of their life (O’Kane, 2008). As the building and playing took place, themes were introduced by the children and were followed up by us. The children explained and played out their experiences. The narrative is established by the interviewer and the child being together ‘at the scene’ of the actual events described by the child. The child’s own story served as the starting point for the conversation as the child’s everyday life unfolds specifically.

Both researchers were present, one leading the conversation and the other writing memos. We taped the interviews and transcribed them literally. The transcribed text and memos are the basis for this article.

### Ethical Considerations

The Norwegian Centre for Research Data approved the study (project nr. 44,076), and the parents gave their written consent allowing their children’s participation in the research group. Even though we had written consent from the parents, we orally informed the children about the project and their role in the research group. We also shared common identity-markers, such as tags with our names, and negotiated common routines and how to proceed in each meeting. We had to be constantly aware of the power imbalance between us and the children, and we informed the children that they could always leave the group or not participate during our visits in the kindergartens. On two occasions our situational ethics allowed children to choose not to participate because they wanted to do something else on one of our research days (Ytterhus & Åmot, 2019).

## Analysis

Methodologically, we used Constructed Grounded Theory (CGT) (Charmaz, 2014) and focused on the children’s doings, discussions, and storytelling. In accordance with CGT we used memo writing from all our visits in the kindergartens and the memos were concurrent with our coding. We followed the recommendations from Charmaz, using initial coding, focused coding, sub-categories, and core categories. The memos have facilitated all our steps in the analysis. The core categories comprise this article’s storyline: “Joy of movement” and “Aspire to social wellbeing”.

**Table 1:** One example of the CGT coding process, clustered together

Initial codes	Focused codes	Core category	Sub-categories
3014. I like to play soccer. My team is Rosenborg (local professional team). (Harold, five years old, without disabilities) 1007. The thing I like best is to play in the woods. (Henry, five years old, with disabilities) 1167. I like sliding on the climbing frame and the seesaw best. (five years old, with disabilities) 122. It’s fun and exciting to slide down the hill (there is only one hill in this kindergarten). (Amy, five years old, without disabilities) 439. I like to play and we do skating and make snow angels. 1120. It is most fun to be in the backyard where I can pick flowers or ride the bike. 415. I like climbing in ropes – one on top to hold on to and one under my feet to balance on.	Inspired by the local sport team Love outdoor physical activity like soccer Like outdoor activity in the local surroundings Like outdoor activity in the local surroundings	Joy of movement	Joy of movement: initiated by children

The rest of the coding and analyzing process were carried out in the same manner as within this table.

## Findings

In our data, the children, who were between four and five years of age, did not use the term “health” very much. In the life-form interviews, we chatted about the day in kindergarten according to topics we as researchers considered relevant so we could understand the children’s experiences of the kindergarten as a health-promoting institution. We were interested in what they *did*, what they liked and did not like, and what they thought about different aspects of their days in kindergarten. Here our focus is on what the children told us about elements of their day-to-day life that refer to healthy spaces and social interaction. They placed health in the context of their daily-life experiences, so when health was mentioned, it was in relation to the professionals’ definitions during meals (healthy food) and organized physical activities (e.g., *mini-moving*).

The children talked a lot about physical activities when asked what they enjoyed doing, and they described places where they were allowed to be active. They also underlined the importance of what we interpret as aspiring to social wellbeing. Two aspects stand out as important when it comes to how children with and without disabilities create healthy spaces and identity in kindergartens. One is the internally driven physical exertion in play and child-controlled activities, and the other is adult-initiated activities. In the data material, social interaction appears to be divided into children’s communities and the role of the staff.

### Joy of Movement: Initiated by Children

In conversations about what they did in kindergarten when they were outdoors, the children focused on what they were interested in when it comes to physical activity. For example, they said things like:

I like to play soccer. My team is Rosenborg (local professional team). (Harold, age five, without disabilities)

In this statement the boy signaled both a sense of local belonging and an often socially appreciated activity consisting of a set of rules and requirements to be able to perform certain skills. The children also talked about the joy of exploring the local environment through physical activity, such as:

The thing I like best is to play in the woods. (Henry, age five, with disabilities)

Heidi, (age five, without disabilities) told us that she had the most fun around the kindergarten’s outdoor area where she could pick flowers, ride a bike, and play.

In this context the children talked about special activities they enjoyed, such as skating or making snow-angels or climbing parallel ropes – one above to hold on to and one below to walk on. Michaela said:

I like sliding on the climbing frame and the seesaw best. (age five years, with disabilities)

Special places in the kindergarten where physical activity can be enjoyed were also mentioned:

It’s fun and exciting to slide down the hill (there is only one hill in this kindergarten). (Amy, age five, without disabilities)

When asked what they miss not having in the kindergarten, one of the boys stated that:

I wish we had a trampoline in the kindergarten. (Benjamin, age five, without disabilities)

When we talked with the children, we wanted to know whether they thought they were able to pursue their preferred activities freely in terms of using their bodies. What we noted then was that the children without disabilities emphasized restrictions and rules relating to physical activity:

We can’t jump on the couch. (Elisabeth, age five, without disabilities)

We can only run and jump and skip outside. (Gemma, age five, without disabilities)

We can jump and run outside, but we can’t shout. (Felix, age five, without disabilities)

Elsie (age five, with disabilities) explains:

We (Edward and I) want to play alone in the doll room, but then the other children run to the staff. They (the staff) get angry with us and tell us that everybody is allowed to enter the doll room ….

The children felt that they had more freedom to use their bodies when they were outdoors than when indoors in the kindergarten. In many of the children’s narratives, the rules and restrictions established by the staff appear to be clear dictates about what is allowed and not allowed when it comes to physical activity:

The kids decide for themselves what they want to play, as long as they don’t run around and make too much noise and stuff like that. (Elisabeth, age five years, without disabilities)

An interesting aspect in this, besides talking about what they liked, disliked, and the many restrictions on their physical activity, is a pattern where the children talked about joyful activities as something *I* like. This is distinct from the references to prohibitions where they mostly described their actions under those conditions as *we, the kids* and as opposites to the staff’s statements.

### Joy of Movement: Staff Initiatives

Excursions or walks, very common occurrences in kindergartens, have various purposes. The children talked about visits to churches, walking past each other’s homes, excursions to the city center, to gyms, and to forests and coastal areas in their local communities.

Sometimes we go on walks from the kindergarten, and then we walk past my house, or we go past other children’s houses. When we walked by my house, we looked at the front of the house, and then there was a picture of me there. (Hanna, age five, with disabilities)

Many children said that the staff decides where to go for a walk and Bjarne (age five, without disabilities) told us that none of the children complain about what the staff suggest. Annie said: I have to go along on an excursion even when I don’t want to. Then the adults say, “Come along then Annie” (age five, with disabilities).

The goal and the purpose of the excursions appear to be strictly regulated by the staff. Only a few of the references to excursions and walks indicated that the children were involved in the decisions, although some of the children said that they liked going on excursions and walks, and that they could play when they have reached their destination.

The children in several of the kindergartens stated that they take part in physical activities led by the staff indoors. Some said that they might sometimes go to the closest indoor sports arena:

I like to be in the sports hall because we can run, jump on the trampoline and play soccer there. (Ava, age five, without disabilities).

One of the kindergartens also had an indoor exercise program for preschoolers (“Mini Moves”) organized in groups, where they perform various exercising activities with instructions from a CD. The staff led these activities When we researchers participated in one of these sessions we observed that the staff made the children aware of their pulse, if it increases or not, and drew attention to the fact that their heart would beat faster when they were active. They were told these were healthy bodily reactions of importance. None of the children mentioned this when we talked to them about their days in kindergarten.

Pervasive elements in what the children told us about their day-to-day lives in kindergarten included activities relating to play and friendship. They explained that they enjoyed playing, that they decided what they liked to play, and that children’s activities in kindergarten are focused on play. They played in the doll corner and play outside.

Indoors in kindergarten we play in the doll corner … we play house (mother-father-children) … and then we play tag. (Elsie, age five, with disabilities)

These are classical role-play activities, and in addition to these, they also played with toys and were physically active. Their play also had restrictions which they saw as being set by the adults:

All the children can join in the play. Only those (children) who run in the walking corridor – they have to go and sit down, that’s what NN (one of the staff) decides. (Emmy, age four, without disabilities)

Physical activity is controlled, and most kindergartens had rules restricting gross motor physical activities indoors.

### Aspire to Social Wellbeing: Children’s Communities

The children in our sample felt that they were generally allowed to decide what to play, as long as they did not make too much noise and were not too rowdy indoors. When outside there is more space for gross motor activities and most of the children talked about the joy of movement and taking part in varied activities.

When they talked about playing, the children also talked about friends and the feeling of joy. They pointed out that they enjoyed playing with friends, that they had friends in the kindergarten, that they were together with friends in many activities, and that the first thing they did when they came to kindergarten was to look for their friends.

My best friends want to play with me. I only have six best friends but counting me it’s really seven! (Emmy, age four, without disabilities)

Other children, with acting-out behavioral and emotional difficulties, disturbed others around them and needed support and supervision from professionals to be tolerated by their peers. Some of these children were defined by their peers as “bad” and even defined themselves as “bad”. Benny, age five, was restless and fidgety, exhibited a short attention span and had a high level of impulsive behavior. When interviewed he referred to himself as someone who likes to scare other children and the other children consistently talked about him as “bad” (?mot & Ytterhus, 2019 ).

In kindergartens located in rural districts the children especially talked about connections between friends in kindergarten and in the local environment:

I usually play with a boy from kindergarten when I’m home. (Hugo, age five, without disabilities)

In Hugo’s kindergarten, there appeared to be a connection between friends in kindergarten and friends at home. When it comes to having friends in the kindergarten, friendships were often across the departments, and the children apparently had the opportunity to visit each other irrespective of what department they were organized into. Some of the children stated that they were currently in the same department as their friends from previous years (for the five-year-olds), even though this was not the case earlier. When considering the creation of social and inclusive spaces for the children who felt safe and confident in their department, the idea of being able to use and be familiar with the entire greater kindergarten area appeared to be important for experiencing oneself as part of the whole.

### Aspire to Social Wellbeing: The Role of the Staff

Children’s communities and social and psychological inclusion have been a major part of the study and have been reported elsewhere (Ytterhus & Åmot, 2021). Here we report on children’s behavior and stories, documenting the need for professional assistance and supervision: health-promoting initiatives. These initiatives had to be introduced when physical closeness constituted problems for some children with behavioral and emotional difficulties. Some children with modest behavioral and emotional difficulties found it hard to connect with their peers, and proximity reflected these difficulties even more:

Sometimes I have nobody to be with, and that makes me a bit sad. Then I tell the staff, and they ask: Why can’t Gemma join you? And then I can join. (age 5, with SEN).

Several children reported that they could rely on the staff when they were in trouble with their playmates, or like Gemma said; she got help when she felt lonely. The children talked about the staff looking after them, that they prepared food, and came to them when assistance and comfort was needed:

The adults don’t play in the kindergarten, they watch so that no one does anything stupid. (Henry, age five, with SEN)

Outside, the adults are checking that all the children are alright! (Gemma, age five, without disabilities)

Beyond this and the fact that the staff decided most things, the children reported that the adults rarely or never participated in their activities or play. When it came to outdoor activities, the staff established clear frameworks for what is and is not allowed, and the staff members were responsible for the children’s security and took them on excursions or walks.

There is also a relatively uniform description of how the staff are absent, in the sense that they did not involve themselves in the children’s play and activities as perceived by the children. The children clearly expressed the wish that the staff could be more involved, as in the words of one child in the Fugleredet (bird’s nest) section:

The adults don’t play. They don’t do anything. I would have liked if they played more. (Florence, age four, without disabilities)

It seems as if the children were telling us how they would like the adults to be co-players and facilitators creating and maintaining a room for interaction and dialog over a particular period of time.

## Discussion

We identified two core categories referring to how children with and without disabilities created healthy spaces and identity in kindergartens: “Joy of movement” and “Aspire to social wellbeing”. To summarize: the children did not have a verbal vocabulary focusing on health issues, but rather on joy and meaningfulness, which means they operated close to Antonovsky’s definition of health. They distinguished between a personalized and individual perspective on activities they liked (“I”), and a collective perspective when describing prohibitions or restrictions as “we’s”. The children in our project presented everyday life as activities and experiences where they distinguish between children’s activities and the tasks assigned by the staff. We will now discuss these elements as (1) healthy activities in everyday life, (2) individual and collective perspectives, and (3) the staff as passive spectators or active facilitators.

Whether kindergartens are to be perceived as health-promoting arenas for children depends on the connection between the individuals and the subsystems of which they are a part. In our context, the interpersonal is about the psychological and biological conditions of the individual child, about the social community they are a part of and the physical environment that surrounds them, influenced by health policy, which is in line with Green et al., (1996).

The Norwegian Directorate for Education and Training (2017) highlights the relevance of social interaction and the awareness of personal limits. In addition to this, our findings show that from children’s perspectives, healthy spaces, indoor and outdoor places, are primarily independent of the individual ability level. These are places and spaces where they described the spontaneous practice of joyful bodily movements and social wellbeing, which is in line with discourses related to children’s development and knowledge of important health issues (Stirrup, 2018).

### Healthy Activities as Everyday Life

The children took their surroundings for granted. However, they were totally aware that their days were structured and that there were places where children, as well as staff, had various positions and tasks. Within these structures the children did not talk about health, physical activity, or exercise. In much the same way as Almqvist et al. (2006) they described their own engagement in preferred activities and actions and their way of managing their everyday-life to achieve wellbeing. From an analytical perspective, we see the link to a global orientation, viewing life as comprehensible, manageable, and meaningful (Antonovsky, 1987). Their descriptions of how they constructed meaning out of their everyday life created their personal way of thinking, being, and acting with an inner trust. In other words, using Antonovsky’s terminology, this can lead to identify, benefit, and use and re-use of the resources at their disposal (Antonovsky, 1987; Lindström & Eriksson, 2010), explaining how they exploit and explore their own physical abilities as they make angels in the snow, climb ropes, or slide down hills. When children enjoyed bodily movements that required physical strength and skills, they seemed to be interrupted in their engagement by the staff’s pointers about heartbeat and pulse. The staff’s efforts to put the children into the preventive-health discourse in an instrumental way did not resonate with the children’s spontaneous way of being where the “I” becomes the “Me” in the next moment (Mead, 1934). One may question if such an instrumental way of practicing health promotion at the individual level strengthens or weakens the children’s identity construction as capable and recognized by their pure presence.

### Individual or Collective Perspectives

In perceiving themselves as an individual in a community, the children were stating two things: (1) In the rural area, local affiliation manifested itself as identification with the local soccer team. They were given time and space to explore their local environment during their days in kindergarten. Both knowledge of the local community’s physical condition and identification with the local soccer team created opportunities for belonging and experiencing wellbeing (Fattore et al., 2009). (2) They perceived themselves as part of a larger whole, as a way of entering a group identity in the kindergarten and in the social community.

All the children, except for Benny and Herold (both age five, with acting out behavioral and emotional difficulties) seemed to distinguish between a personalized and individual perspective on activities they liked: as either *I*’s or describing themselves as a part of a group, as *we*’s. When they described what they found to be fun, what they liked to do within physical activities, many of the statements were characterized by an *I*, a story in which they themselves were interested (for example, when Hanna said that *we* go for a walk), then it is home to an *I*.

At the same time, it is striking that in many of the statements where the children talked about a number of prohibitions and restrictions regarding physical activity, they presented themselves as a *we* and not as an individual child. In relating to adult authority and symbolic power, the children presented a collectivistic thinking when it comes to being in kindergarten. The individual child is the actor, but when the individuals face the staff’s authority and symbolic power, the subject becomes a “we”. This shows that they related to the staff’s restrictions and requirements with an understanding that is approximately “the staff” versus “we, the children”. In this way the *I* represents the spontaneous, not necessarily the predictable reactions of the child (Mead, 1934, 1998). Merely the fact that the children are present in a social action gives meaning. It is interesting that the interpretations of prohibitions and restrictions created a common language and thus, also common symbols that gave common meaning to the children. The social process stimulated by the others (the staff) with its restrictions seemed to create a social self by relating to the situation and the specific expectations for social behavior. According to Mead the internal awareness of the social situation and the socialization process created a space for “us” (the children) and “them” (the staff).

### The Staff as Passive Spectators or Active Facilitators

The staff seemed in many ways to represent the personalized factor to ensure a structured, predictable, and organized everyday life. The children described the sanctions and the restrictions as explanations of the structure and organization. In this way we can describe the staff as important for the children to ensure that the stimuli from the internal and external environments were structured, predictable, and explicable, and thus, created a sense of coherence (Eriksson, 2017). On the other hand, we can question if their use of such concepts as healthy activities and healthy food are specific ways of using their symbolic power in line with the existing health discourse in their attempt to implement the public policy, instead of putting an effort into the recognition of the children’s presentation of everyday life as activities and experiences. The children seemed to miss the staff’s engagement in their activities. Engagement in the children’s joy of movement in everyday life may be more important, and provide a wider opportunity set, than the staff leading through “Mini Moves”. Demands posed by the natural stimuli offer challenges worthy of investment and engagement (Eriksson, 2017). The children wanted the staff to be players and facilitators.

Healthy spaces in our study arose when, independent of their abilities, the children acted and played at places in kindergarten and in ways that made it possible for all of them to understand what was going on. This, as well as knowing how to behave to keep the preferred activities going in a manner that makes the activity meaningful, helped the children to experience a sense of coherence.

Nevertheless, healthy activities as described here are constructed in the children’s daily life as here-and-now moments put into play by the children. When it comes to the children’s identity constructions, for example Benny’s and other children’s descriptions of him as “bad”, it seems necessary to underline the staff’s responsibilities to ensure and aspire wellbeing. Benny, and children who need help to find positive structures and constructive meanings of *I* are entitled to individual guidance so they can figure out how to act to be included as a part of the children’s *we* and not as *the other.* This is an example of social space that influences risks for unhealthy constructions of the self (Mead, 1934; Mæland, 2020b).

## Conclusions

Children created healthy spaces in kindergarten by focusing on play, interactions, and identity construction as they described everyday routines and daily content. The children called for the staff’s engagement in play and activities and they relied on support from the staff when needed. Individual support presupposes active engagement from the staff in the children’s activities as a way of recognizing their ongoing discourse of joy and wellbeing as a comprehensible, manageable, and meaningful way of living. The children illustrated the importance of the interaction between individuals and their context, which requires efforts to change practice on an organizational level. The children required actions originating on the organizational level where structures open for an increase in the staff’s active participation in the children’s activities. This requires spaces for professional engagement and reflections on the staff’s role and obligations in everyday life in kindergartens.

## Limitations

We had control over the recruitment of disabled children, therefore, only one child was diagnosed with disabilities that potentially led to social difficulties in one kindergarten department. However, as we left the final choice of the recruitment of non-disabled children to the staff, even when matching for gender and age, we cannot be certain as to how this situation influenced the interaction within the research groups. Additionally, this study is based on a small sample and on the individual level. Further analyses on the structural level when it comes to health promotion and children’s everyday life in kindergarten will be necessary to gain greater insight into the stakeholder’s and staff’s perspectives.

## Electronic supplementary material

Below is the link to the electronic supplementary material.

Supplementary Material 1

## References

[CR1] Ahlcrona MF (2012). The Puppet’s Communicative Potential as a Mediating Tool in Preschool Education. International Journal of Early Childhood.

[CR414] Almqvist, L., Hellnäs, P., Stefansson, M., and Granlund, M. (2006). ‘I can play!’ Young children’s perceptions of health. Pediatric Rehabilitation, 9(3), 275–284. 10.1080/13638490500521303 10.1080/13638490500521303 17050405

[CR44] Åmot, I. (2014). Barn med samspillsvansker og ansattesdilemma relatert til retten til medvirkning i barnehagen [Children with interaction difficulties and employees' dilemmarelated to the right to participation in kindergarten], (pp 341). Trondheim. Norwegian University of Science andTechnology.

[CR43] Åmot, I. & Ytterhus, B. (2019). Spesialpedagogikk i barnehagen. [Special education in kindergarten]. In E. Befring, K. A. B. Næss & R. Tangen (Eds.),* Spesialpedagogikk*. (6thed.) (pp 499 - 614). Cappelen Damm Akademisk.

[CR2] Almqvist L, Hellnäs P, Stefansson M, Granlund M (2006). ‘I can play!’ Young children’s perceptions of health. Pediatric Rehabilitation.

[CR3] Andenæs A (1991). Fra undersøkelsesobjekt til medforsker? Livsformintervju med 4–5 åringer. [From research-object to research-collaborator? Life-form interviews with 4 to 5-year-old children]. Nordisk psykologi.

[CR4] Antonovsky, A. (1987). *Unraveling The Mystery of Health - How People Manage Stress and Stay Well.* Jossey-Bass Publishers.

[CR6] Barneombudet (2013). *Eksperthåndbok. Kort innføring i å holde ekspertmøter og opprette ekspertgrupper.* http://barneombudet.no/wp-content/uploads/2013/09/eksperthandbok.pdf

[CR7] Cammisa M, Montrone R, Caroli M (2011). Development and results of a new methodology to perform focus group with preschool children on their beliefs and attitudes on physical activity. International Journal of Pediatric Obesity.

[CR8] Carson V, Lee EY, Hewitt L, Jennings C, Hunter S, Kuzik N, S.,Tremblay (2017). Systematic review of the relationships between physical activity and health indicators in the early years (0–4 years). Bmc Public Health.

[CR9] Charmaz, K. (2014). *Constructing Grounded Theory*. Sage

[CR10] Eriksson, M. (2017). The Sense of Coherence in the Salutogenic Model of Health. In M. B. Mittelmark, S. Sagy, M. Eriksson, et al. (Eds.), *The Handbook of Salutogonensis*. Springer. doi: 10.1007/978-3-319-04600-6_11 28590620

[CR11] Fattore T, Mason J, Watson E (2009). When children are asked about their well-being: towards a framework for guiding policy. Child Indicator Research.

[CR12] Frerichs L, Intolubbe-Chmil L, Brittin J, Teitelbaum K, Trowbridge M, Huang TTK (2016). Children’s Discourse of Liked, Healthy, and Unhealthy Foods. Journal of the Academy of Nutrition and Dietetics.

[CR13] Green LW, Richard L, Potvin L (1996). Ecological Foundations of Health Promotion. American Journal o f Health Promotion.

[CR14] Holt-Lunstad J, Smith TB, Layton JB (2010). Social Relationships and Mortality Risk: A Meta-analytic Review. Plos Medicine.

[CR15] Klein D, Manz K, Ferrari N, Strüder H, Graf C (2015). Effects of health promotion projects in preschools on body mass index and motor abilities. The Journal of sports medicine and physical fitness.

[CR16] Kobel, S., Wartha, O., Wirt, T., Dreyhaupt, J., Lämmle, C., Friedemann, E. M., & Steinacker, J. M. (2017). Design, Implementation, and Study Protocol of a Kindergarten-Based Health Promotion Intervention. *BioMed Research International, 2017*, 9. doi:10.1155/2017/4347675 10.1155/2017/4347675 PMC5338306 28303253

[CR17] Lindström, B., & Eriksson, M. (2010). *The Hitchhiker’s Guide to Salutogonesis. Salutogenic pathways to health promotion. Folkhälsan research center*. Health promotion research.

[CR18] Lloyd B, Buffett KM, Innes-Hughes C, Jackson D, Qi J, Powell L (2017). Supported Playgroups for Health Promotion Activity for Healthy Eating and Active Living: A Social Ecological Perspective. Australasian Journal of Early Childhood.

[CR19] Mead, G. H. (1934). *Mind, self, and society from the standpoint of a social behaviorist*. University of Chicago Press.

[CR20] Mead, G. H. (1998). *Å ta andres perspektiv: grunnlag for sosialisering og identitet: George Herbert Mead i utvalg [Taking the other’s perspective; foundation for socialization and identity: Selected readings of George Herbert Mead]*. Abstrakt forlag.

[CR21] Ministry of Education and Research (2005). *Kindergarten Act*. Act no. 64 of June 2005 relating to Kindergartens. Retrieved October 1, 2018. Norwegian Ministry of Education and Research. https://www.regjeringen.no/globalassets/upload/kd/vedlegg/barnehager/engelsk/act_no 64_of_june_2005_web.pdf.

[CR22] Ministry of Health and Care (2012). The Public Health Act (Folkehelseloven).

[CR23] Mæland, J. G. (2020a). På jakt etter sykdommers sosiale røtter (Hunting the social roots of illnesses). In Mæland J.G. (Ed), *Sykdommers sosiale røtter. *Gyldendal: 16–37.

[CR24] Mæland, J. G. (2020b). Jo mere vi er sammen… Sosial kontakt og samhold beskytter mot sykdom [The more we are together… Social contact and interaction protects against illness]. In: John Gunnar Mæland (ed) *Sykdommers sosiale røtter *(pp. 103–123). Gyldendal.

[CR25] Mæland, J. G. (2016). *Forebyggende helsearbeid: folkehelsearbeid i teori og praksis [Health prevention: public health work in theory and practice]* (4th ed.). Universitetsforlaget.

[CR26] Normandeau S, Wins I, Jutras S, Hanigan D (1998). A description of 5- to 12-year old children’s conception of health within the context of their daily life. Psychology & Health.

[CR27] Norwegian Directorate for Education and Training (2017). *Framework Plan for Kindergartens. Contents and tasks.* Oslo: The Norwegian Directorate for Education and Training. Retrieved June 2020 from https://www.udir.no/globalassets/filer/barnehage/rammeplan/framework-plan-for-kindergartens2-2017.pdf

[CR28] O’Kane, C. (2008). The development of participatory techniques. In P. Christensen, & A. James (Eds.), *Research with children: Perspectives and practices* (pp. 125–155). Routledge.

[CR29] Sando OJ (2019). The outdoor environment and children’s health: a multilevel approach. International Journal of Play.

[CR30] Sando OJ (2019). The physical indoor environment in ECEC settings: children’s well-being and physical activity. European Early Childhood Education Research Journal.

[CR31] Sando, O. J., & Mehus, I. (2019). Supportive indoor environments for functional play in ECEC institutions: a strategy for promoting well-being and physical activity? *Early Child Development and Care*, 1–12. doi:10.1080/03004430.2019.1651305

[CR32] Sandseter EBH, Seland M (2016). Children’s Experience of Activities and Participation and their Subjective Well-Being in Norwegian Early Childhood Education and Care Institutions. Child Indicators Research.

[CR33] Sandseter EBH, Seland M (2017). 4–6 year-old Children’s Experience of Subjective Well-being and Social Relations in ECEC institutions. Child Ind Res.

[CR34] Statistics Norway (2020). https://www.ssb.no/utdanning/faktaside/utdanning, Retrieved 15 June, 2021

[CR35] Stirrup, J. (2018). ‘*How do you feel? What is your heart doing?’… It’s jumping’: the body and health in Early Years Education.* Sport, Education and Society *23*(6), 547–562 Doi *10.1080/13573322.2016.1259613*

[CR36] United Nations (2015). The Sustainable Development Goals: https://sdgs.un.org/goals

[CR37] Wagner N, Meusel D, Höger C, Kirch W (2005). Health promotion in kindergarten children: an assessment of evaluated projects in Germany. Journal of Public Health.

[CR38] World Health Organisation (1948). *Defintion of health.* Retrieved April 7, 2020 from https://www.who.int/about/who-we-are/constitution

[CR39] World Health Organization. (1978). *Declaration of Alma-Ata* (pp. 6–12). USSR. September 1978.

[CR40] World Health Organization (1998). *Health Promotion.* Retrieved June 28, 2021 from https://www.who.int/health-topics/health-promotion#tab=tab_1

[CR42] Ytterhus, B. & Åmot, I. (2021). Kindergartens:Inclusive Spaces for All Children? *International Journal of Inclusive Education*, 10.1080/13603116.2021.1950976

[CR41] Ytterhus, B. & Åmot, I. (2019). Barn med og uten funksjonsnedsettelser og deltagelse i forskergrupper ? når oghvordan kan ulike kvalitative forskningsmetoder få alle barn til ? delta i forskning? *Tidsskrift for Nordiskbarnehageforskning*, *18*(9), 1–15. 10.7577/nbf.3286

