# Supplementary material for: Health Promotion and Identity Construction in Norwegian Kindergartens – A Qualitative Study on Children with and without Disabilities
Source: Early Child Educ J. 2022 Oct 10:1–11. Online ahead of print. doi: 10.1007/s10643-022-01382-7 (PMC9549454; doi:10.1007/s10643-022-01382-7)
Supplement: Supplementary file 1 — Supplementary Material 1 [file 10643_2022_1382_MOESM1_ESM.docx]

## Abstract

Health promotion and identity construction in Norwegian kindergartens – a qualitative study on children with and without disabilities

This article focus on how children independent of abilities create healthy identity and spaces in kindergarten, and is based on a qualitative CGT-study carried out in Norwegian Kindergartens. Data material is Life-form interviews with 24 children, with and without disabilities. Children placed health in the context of their daily-life experiences. They underline the importance of aspiring social well-being and create healthy spaces through internally driven physical exertion and child-controlled activities. Children describe how they relate to the staff’s restrictions and legal requirements as "the staff" versus "we, the children». They advertise for staff’s engagement in play and activities.
